# ﻿A new noctuid genus and species (Lepidoptera, Noctuidae, Amphipyrinae, Psaphidini, Triocnemidina) from New Mexico and Texas, United States of America

**DOI:** 10.3897/zookeys.1200.117772

**Published:** 2024-05-08

**Authors:** Lars G. Crabo

**Affiliations:** 1 Adjunct Faculty, Washington State University, Pullman, Washington, USA Washington State University Pullman United States of America

**Keywords:** Chihuahuan desert, DNA barcode, key, new combination, new genus, new species, owlet moth, systematics

## Abstract

*Poolea***gen. nov.** is described for two noctuid species from southwestern United States: *Pooleagrandimacula* Barnes & McDunnough, **comb. nov.**, previously in *Oxycnemis* Grote, and *Pooleapsaphidoides***sp. nov.***Poolea* is compared to *Oxycnemis* (Amphipyrinae, Psaphidini, Triocnemidina) and is retained in the same subtribe. Adult moths and male and female genitalia of *Poolea* species are illustrated along with those of *Oxycnemisadvena* Grote, the genus type species. Pertinent recent taxonomic changes to Amphipyrinae classification are reviewed.

## ﻿Introduction

An undescribed noctuid species that superficially resembles several species of *Psaphida* Walker but is congeneric with “*Oxycnemis*” *grandimacula* Barnes & McDunnough (Amphipyrinae, Psaphidini) was collected during the spring of 2020 in New Mexico, USA. “*Oxycnemis” grandimacula* and the newly found species differ significantly from *Oxycnemisadvena* Grote (Psaphidini, Triocnemidina), the type species of *Oxycnemis* Grote, and cannot be assigned satisfactorily to any extant genus. Description of this species and a genus for it and its congener “*Oxycnemis*” *grandimacula* are the main purposes of this communication.

The noctuid subfamily Amphipyrinae has a long and tumultuous history, which is reviewed partially in the Systematics section. Significant contributions to the currently accepted classification were advanced by Poole (1995, 2022), including on the unpublished website Nearctica.com–cited here as 2022, the year it was accessed, although it was written at unknown times at least a decade or two earlier. On this website, “*Oxycnemis*” *grandimacula* and an undescribed congener, almost certainly the same as the New Mexico moth, were assigned to an undescribed genus in Triocnemidina. Poole did not name the genus nor the species, and neither has been described formally until now. Nonetheless, Poole’s (1995, 2022) work provides a solid morphological framework for the subtribe, as well as pointing out several other taxa in need of further study.

## ﻿Material and methods

Wing pattern and genitalia structure terminology follow Lafontaine (2004). Forewing length from base to apex, excluding the fringe, is measured to the nearest half millimeter.

Genitalia were prepared by the methods of Hardwick (1950) and Lafontaine (2004). The detached abdomen was macerated in hot 10% potassium hydroxide for 20–30 minutes. Dissection was performed in water followed by hardening in 95% isopropyl alcohol. Male vesicae and female bursae were inflated. Preparations were stained with orcein (Sigma Chemical Company, St. Louis, Missouri) and mounted in Euparal (Bioquip Products, formerly of Rancho Dominguez, California). Spacers were used to elevate the cover glasses from the slides in order to preserve the three-dimensional shape of the inflated structures.

The 658 base pair “barcode” region of mitochondrial cox1 *mt* DNA (hereafter “DNA barcode”) was used to assess the taxonomic placement of species and genera. Legs from dried specimens submitted to the Barcodes of Life Data System (BOLD) at the University of Guelph (Ontario, Canada) were analyzed by standard extraction, amplification, and sequencing protocols (Hebert et al. 2003). Barcodes were compared to existing material at BOLD as implemented at barcodinglife.org. The seven-digit Barcode Index Numbers (BIN) (Ratnasingham and Hebert 2013) are assigned by BOLD.

The distribution map was made using SimpleMappr (http://simplemappr.net).

### ﻿Repository abbreviations

**CNC** Canadian National Collection of Insects, Arachnids, and Nematodes, Ottawa, Ontario, Canada

**CSUC** Colorado State University Collection, Fort Collins, Colorado, USA

**DLWC** Dave Wikle personal research collection, San Marino, California, USA

**JVC** Jim Vargo personal research collection, Mishawaka, Indiana, USA

**LGC** Lars Crabo personal research collection, Bellingham, Washington, USA

**NMNH** National Museum of Natural History, Smithsonian Institution, Washington D.C., USA

## ﻿Systematics

Triocnemidina was described by Poole (1995) originally as a tribe of Psaphidinae and was revised informally on Nearctica.com (Poole 2022). This subtribe is defined by a combination of adult characters (Poole 1995, 2022): head with unmodified frons and simple or weakly serrate male antenna; prothoracic tibia with a strong clawlike seta continuous with a knifelike ridge along the tibia, often with an accessory claw (Fig. 10); hindwing cross vein mdc with concave anterior segment and laterally-directed angle at strong M2; abdominal spiracles with weak distal wall and well-developed dorsal lever; male A7 tergum large, thickly sclerotized, with weakly bilobed distal margin; male valve simple, with porrect spinelike ampulla of the clasper arising near the ventral margin, and hairlike setae but no corona on the unmodified cucullus (Figs 5a, 6a); male phallus with tubular vesica bearing one or two patches of spinelike setae set on crenulate bands (Figs 5b, 6b); female bursa copulatrix simple with or without segmented bandlike signum (Figs 8, 9); and external tympanum with large hood with large ovate or rectangular bulla.

Triocnemidina is a small subtribe mostly from deserts of southwestern United States and Mexico. Poole included in it eight described and three undescribed species in seven genera: four named genera (*Crimona* Smith, *Oxycnemis*, *Policocnemis* Benjamin, and *Triocnemis* Grote), one described subsequently (*Unciella* Troubridge, 2008), and two that have remained undescribed (“Triocnemidina New Genus 1,” described below, and “Triocnemidina New Genus 3” for “*Unciella*” *flagrantis* (Smith)). Each of these genera contains only one or two species. Several changes to the subtribe have occurred since it was first proposed: Triocnemidini was removed briefly from Psaphidinae based on pupa morphology and larva biology (Kitching and Rawlins 1999), but reassigned to it by Fibiger and Lafontaine (2005); Psaphidinae was subsumed as a tribe of Amphipyrinae based on larva morphology (Wagner et al. 2008); *Unciella* was described; and *Oxycnemis* was found to be a paraphyletic assemblage of genera belonging in two subfamilies (Keegan et al. 2019). In the latest checklist of Pohl and Nanz (2023) “*Oxycnemis*” *gracillinea* Grote is moved provisionally to *Sympistis* Hübner (Oncocnemidinae) along with “*Oxycnemis” acuna* (Barnes), and *Hemigrotellaargenteostriata* Barnes & McDunnough is included in Triocnemidina for the first time. The latest changes are based on the seminal studies of Keegan et al. (2019, 2021) which are discussed further below.

### ﻿ Poolea gen. nov.

Taxon classificationAnimaliaLepidopteraNoctuidae

2AB4A23D-30F7-5056-B403-BF75835AB8DC

https://zoobank.org/70148D54-09C8-4489-807C-11739AAAAB08

#### Type species.

*Oxycnemisgrandimacula* Barnes & McDunnough.

#### Gender.

Feminine.

#### Diagnosis.

Adults (Figs 2, 3) are medium-sized moths (FW length 11.5–16.0 mm) with gray forewings with large black-outlined spots, reniform spots weakly quadrate. They superficially resemble *Psaphida* or *Pseudocopivaleria* Buckett & Bauer (Amphipyrinae, Psaphidini, Psaphidina) but can be distinguished by their modified foretibial setae, a strong claw continuous with a knifelike ridge along the tibia and a distinct smaller lateral accessory claw in *Poolea* (Fig. 10), simple in the other genera. This claw shape also distinguishes *Poolea* from superficially similar species of *Sympistis*, which lack an accessory claw. The few Triocnemidina species that resemble *Poolea* in size and shape, particularly *Triocnemissaporis* Grote and *Unciellaprimula* Smith, are predominantly whitish or yellow tan instead of gray.

Male valves of *Poolea* (Figs 5a, 6a) are similar to those of *Triocnemis*, simple with a moderate sacculus and a narrow thornlike clasper of the ampulla arising near the ventral margin slightly past the midpoint of the valve. However, the uncus of *Poolea* is unique in Triocnemidina, straight and rodlike, oriented slightly dorsad at base, with slight subapical swelling and short fingerlike apex with a slight downward hook. That of *Triocnemis* is curved, tapering evenly from base to apex. The vesica of *Poolea* (Figs 5b, 6b) is bent subbasally and bears a single long band of spinelike setae of variable lengths.

The female genitalia of *Poolea* (Figs 8, 9) are also distinctive. The bursa copulatrix is asymmetric with the ovoid corpus bursae joined obliquely to the ductus bursae and extending posterior and leftward. The corpus bursae lack signa, and the ductus seminalis is joined to the conical posterior end. Most of the ductus bursae is sclerotized and has longitudinal rugae, with a membranous short posterior segment adjacent to the weakly sclerotized ostium bursae. The anterior apophysis is longer than the posterior apophysis. The ovipositor lobe is padlike.

**Figures 1–3.**
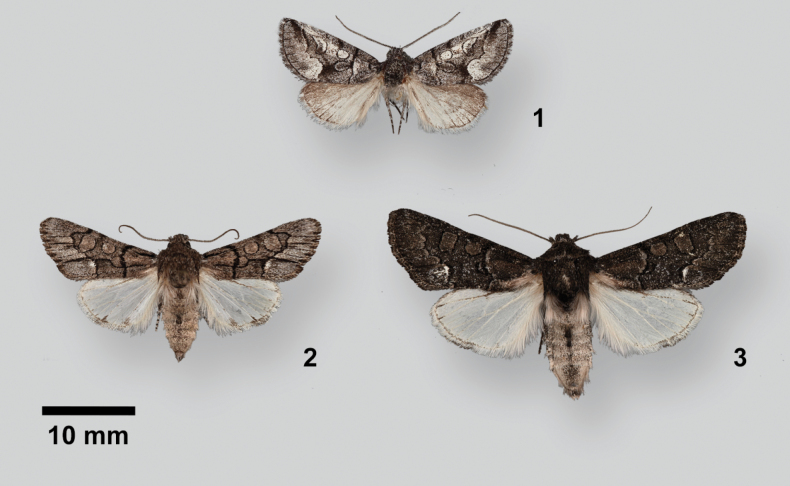
Adult males **1***Oxycnemisadvena* (abdomen removed), USA, Arizona, Maricopa County, Cave Creek **2***Pooleagrandimacula*, USA, Texas, Brewster County, Terlingua Ranch **3***Pooleapsaphidoides*, USA, New Mexico, Otero County, High Rolls.

**Figures 4–6.**
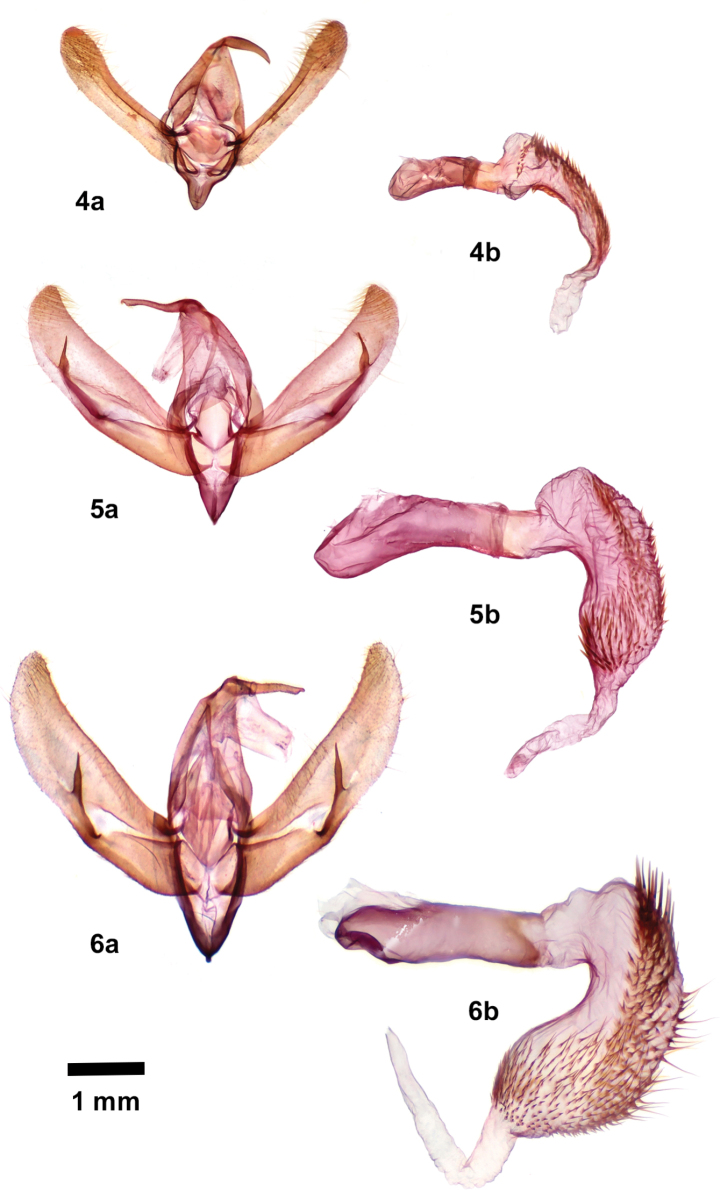
Male genitalia, valves (**a**) and phallus (**b**) with everted vesica **4***Oxycnemisadvena***5***Pooleagrandimacula***6***Pooleapsaphidoides*

**Figures 7–9.**
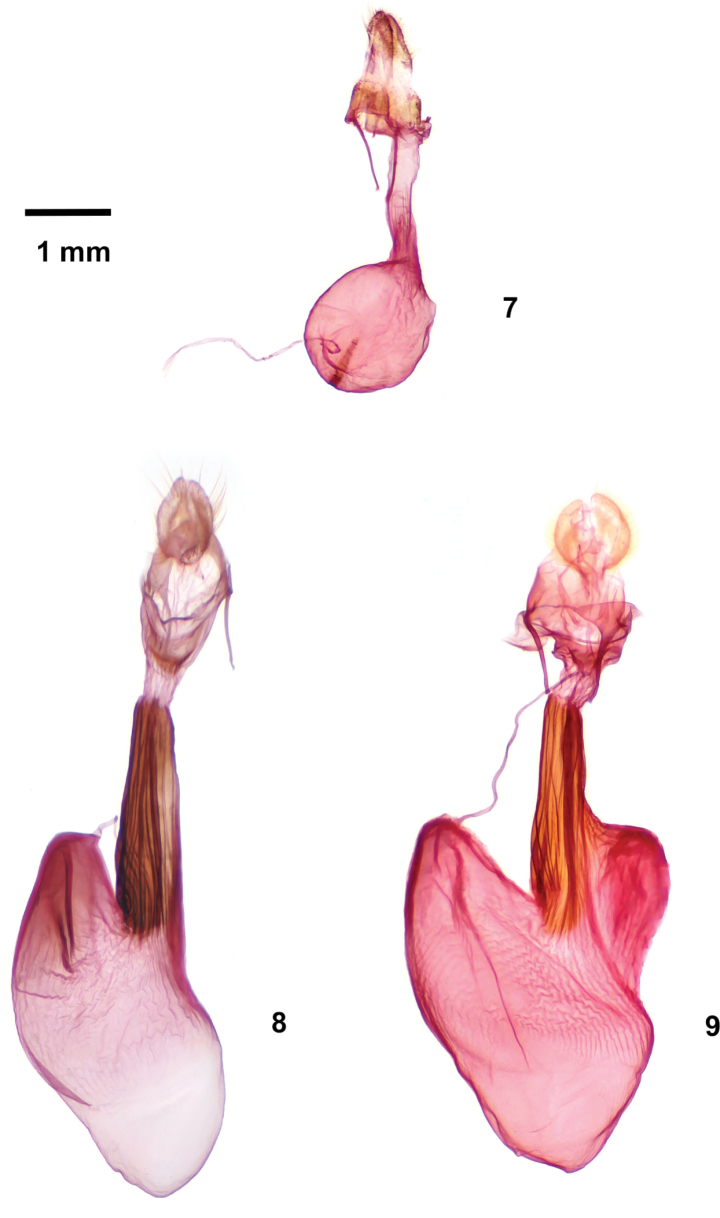
Female genitalia **7***Oxycnemisadvena***8***Pooleagrandimacula***9***Pooleapsaphidoides*

*Pooleagrandimacula* was described as an *Oxycnemis*, type species *O.advena* (Fig. 1). It remained in the genus until the most recent North American checklist (Pohl and Nanz 2023). A footnote by section author B. C. Schmidt states that there is no named genus for *grandimacula*, and it is placed in “*Oxynemis*” with quotation marks to distinguish it from *Oxycnemis* sensu stricto. *Poolea* is significantly structurally distinct from *Oxycnemis* in both sexes. The male valve of *Oxycnemisadvena* (Fig. 4a) is a nearly featureless strap with a rounded apex, simpler than those of *Poolea*, lacking a distinct sacculus and bearing a small nubbinlike triangular ampulla. The uncus of *Oxycnemis* is curved, with well-developed sacculus and clasper. The vesica of *Oxycnemis* (Fig. 4b) is nearly straight with two bands of setae; that of *Poolea* is bent with a single band of setae. The female corpus bursae of *Oxycnemis* (Fig. 7) is entirely membranous, volumetric-flask-shaped with a spherical corpus bursae and tubular ductus bursae. Unlike *Poolea* it has a long segmented signum and the ductus seminalis is at the anterior end. The papilla analis of *Oxycnemis* is conical rather than padlike.

DNA barcodes also clearly support that *Poolea* and *Oxycnemis* are distinct genera (Fig. 11). The two *Poolea* species differ by over seven percent from *O.advena*, a larger difference than between most other genera in the subtribe.

#### Description.

***Adult*.** Sexes superficially similar, of medium size (forewing length 11.5–16.0 mm). Vestiture of dorsal head and dorsal thorax black, grayish tan, and white long predominantly simple and scattered apically forked or spatulate serrate scales, concolorous with dorsal forewing. *Head* – Antenna of both sexes filiform, setose ventrally, dorsal scales small. Eye normal size, lacking hairs. Frons convex, lacking tubercle, scales thin, mesially directed. Labial palpus relatively short, reaching mid eye, vestiture of short straplike lateral and long piliform ventral scales. Haustellum normal. *Thorax* – Prothoracic collar with weak mesial crest and subbasal black transverse line; tegula with weak black line near medial margin; meso- and metathorax with loose tufts of curly spatulate glossy black scales, posterior tufts largest. *Legs* – Foretibia with distal stout trifurcate clawlike seta, apical process a large stout beaklike spine projected along foretarsus, middle process a small outer claw directed slightly laterally, basal process a knifelike ridge along tibia; mid- and hindlegs unmodified; ventral tarsi segments with scattered spiniform setae between haphazard inner and outer rows. *Wings* – Forewing elongate, length 2.3–2.6× width, with bluntly pointed apex and smoothly convex outer margin with slight indent at Cu2; dorsal scales smooth, short, slightly convex distally with finely crenulate margin; ground light to charcoal gray or dark olive, darkest in medial area outside spots; transverse lines and spot outlines black, single; basal line absent; antemedial line smooth, thick, excurved strongly from anterior margin to fold, less prominently thence to posterior margin, bordered medially by uniform pale line; medial line absent or a dark smudge posterior to orbicular spot; postmedial line nearly parallel to outer margin, drawn slightly basad in fold, moderately scalloped between veins, posterior segment thickest, bordered laterally by gray to white, palest and widest in fold; subterminal line absent; terminal line thin, preceded by triangular dark marks between veins in one species; spots large, filled with wing base color or slightly lighter shade; orbicular spot ovoid; claviform spot semicircular; reniform spot quadrate with rounded corners, slightly concave medially and laterally; fringe weakly checkered ground color or brown with slightly darker gray; scattered black dashes on veins (one species), strongest in fold in medial area and on Cu2 distal to postmedial line. Hindwing M2 strong, similar to mdc but weaker than M1 and M3, crossvein mdc concave anterior to M2, angled distad at M2, thence perpendicular to M3; dorsum white, few dark gray scales at tornus, on terminal line and distal veins, especially Cu2; fringe white with scattered gray scales. *Abdomen* – Lacking basal scent brushes and associated structures. A7 tergum large and strongly sclerotized, distal margin weakly bilobed. Spiracles with degenerate distal wall and long dorsal lever, resembling upper component of a question mark. Scales gray tan, weak darker dorsal tufts on segments 1, 3, and 4. *Male genitalia* – Uncus base directed posterior and slightly dorsad, with slight subbasal dorsad and subapical ventrad bends, cylindrical with slight subapical swelling bearing few short setae on flat venter, tip tapered to small downward hook with blunt tip. Juxta shield shaped, taller than wide. Valve nearly even width, widest near midpoint, length 3.6–4.0× width, with rounded slightly upturned apex bearing a mesial patch of downy setae; sacculus moderately strong, 0.5× valve length and 0.5× valve width at midpoint, tapered evenly from base to apex; clasper base a sclerotized bar near ventral margin distal to sacculus, ampulla base arising near valve midpoint (one species) or at outer ¾ (one species), perpendicular to valve, nearly reaching dorsal margin, thornlike, narrow, tapered to thin acute tip; digitus absent. Phallus tubular, length ~ 6× minimum width; vesica with broad basal dorsal bulge and subbasal 90–135° bend ventrad and anterior, about as long and slightly wider than phallus, widest near apex; mid and distal surface covered by broad band of multiple variable-length spiniform cornuti extending from left posterior surface distal to bulge to apex where it spirals ¾ around circumference, cornuti longest at mid vesica, gradually decreasing distally, subbasal cornuti longer and thicker than adjacent cornuti in one species. *Female genitalia* – Corpus bursae unisaccate, asymmetrically ovoid with membranous bulbous anterior end and thicker broadly conical thicker posterior end with ductus seminalis at apex, length about 1.5× width, lacking signa. Ductus bursae 5× segment A8 length, tubular (one species) or with anterior broad bulge rightward (one species), constricted slightly at junction of anterior ¾ and posterior ¼, short posterior segment membranous, anterior segment sclerotized with longitudinal rugae, joined obliquely to right posterior corpus bursae; ostium bursae weakly sclerotized. Papilla analis soft, padlike, covered with uniform thin piliform setae. Segment A8 short, length 2.7× width, dorsum longest; apophyses relatively short, posterior apophysis 1× segment A8 length, anterior apophysis 1.5× posterior apophysis.

#### Etymology.

The genus name honors Robert “Bob” Poole for his contributions to the systematics of Amphipyrinae.

#### Distribution and ecology.

*Poolea* species occur in southwestern United States and Mexico, predominantly in the Chihuahuan desert region. All United States records are from Arizona, New Mexico, and Texas. Adults fly in arid shrubland from spring to fall (one species) or spring (one species). The early stages are unknown.

The large foretibial claw of *Poolea* is probably an adaptation to allow moths to escape from the pupal chamber in hard desert soils and dig their way to the ground surface.

##### ﻿Key to adults of *Poolea* and *Oxycnemisadvena*

**Table:** 

1	Foretibial claw with strong lateral accessory claw (Fig. 10); male valve with long thornlike ampulla of clasper (Figs 5a, 6a); female ductus bursae strongly sclerotized with longitudinal rugae (Figs 8, 9)	***Poolea* 2**
–	Foretibial claw simple, lacking accessory claw; male valve with diminutive triangular ampulla of clasper (Fig. 4a); female ductus bursae membranous (Fig. 7)	***Oxycnemisadvena***
2	Smaller species (forewing length 11.5–15 mm); forewing medium gray with distinct longitudinal black dashes (Fig. 2); male valve with ampulla of clasper arising on distal valve closer to base of cucullus than to sacculus (Fig. 5a); female ductus bursae tubular, broadest anteriorly (Fig. 8)	***P.grandimacula***
–	Larger species (forewing length 14–16 mm); forewing charcoal gray to dark olive without dashes (Fig. 3); male valve with ampulla of clasper arising on mid valve near sacculus (Fig. 6a); anterior female ductus bursae broadly convex toward right (Fig. 9)	***P.psaphidoides***

### ﻿ Poolea grandimacula

Taxon classificationAnimaliaLepidopteraNoctuidae

(Barnes & McDunnough) comb. nov.

0DDB1ADE-7708-544E-AEC2-267010E8502C

Figs 2 , 5a, b , 8

Oxycnemis grandimacula Barnes & McDunnough, 1910
Oxycnemis extremis Barnes & McDunnough, 1913

#### Type material.

*Oxycnemisgrandimacula* was described from Redington, Arizona (NMNH) (holotype examined from photograph). *Oxycnemisextremis* was described from two syntypes, one from Brownsville and the other from San Benito, Texas (NMNH) (syntypes examined from photographs). The Brownsville syntype is darker than most *P.grandimacula*, but its forewing pattern is otherwise typical of this species.

#### Diagnosis.

*Pooleagrandimacula* is distinguished by several longitudinal black lines on the forewing antemedial, medial, and postmedial areas which *Pooleapsaphidoides* lacks. *Pooleagrandimacula* tends to be smaller than *P.psaphidoides* (FW length of *P.grandimacula* 11.5–15 mm; *P.psaphidoides* 14–16 mm) and the FW is lighter gray with more discernible pattern. The ampulla of the clasper of the male valve arises more distally in *P.grandimacula* than in *P.psaphidoides* as described in the key. Females can be distinguished by the shape of the ductus bursae, tapered evenly in *P.grandimacula* and bulging rightward near the junction with the corpus bursae in *P.psaphidoides*.

#### Distribution and ecology.

*Pooleagrandimacula* occurs in southwestern United States in Arizona, New Mexico, and western and southern Texas. Poole (2022) also examined a specimen from Nuevo León, Mexico. Adults fly in open xeric habitats and have been collected as early as March and as late as October, suggesting multiple broods. The early stages are unknown.

This species and *P.psaphidoides* are sympatric in southern New Mexico, including at White Sands National Park, Otero County.

### ﻿ Poolea psaphidoides sp. nov.

Taxon classificationAnimaliaLepidopteraNoctuidae

71B20473-CF78-5766-BFC5-8E5D6CB24744

https://zoobank.org/F5762B69-E68A-4D49-8C71-83415FF96CA2

Figs 3 , 6a, b , 9 , 12

#### Type locality.

USA: New Mexico: Otero County: High Rolls, Steep Hill Rd., 32.9534, -105.8817, 1725 m.

#### Type material.

***Holotype*, male.** USA: New Mexico: Otero County: High Rolls, Steep Hill Rd., 32.9534, -105.8817, 1725 m, 11 III 2020, L. G. Crabo leg. / [Crabo genitalia slide] 651 male. CNC. ***Paratypes*.** 15 m, 3 f. USA: New Mexico: Eddy County: 32.272, -104.602, 4200’ [1280 m], 20 III 2021, J Vargo (8 m 2 f); Carlsbad, 32.4420, -104.2776, 1060 m, 4 III 2024, David Heckard / DLWC 011521 (1 m); Otero County: same data as holotype (3 males); same data as holotype / BOLD_F8 CHLC0068 (1 male); same data as holotype / BOLD_F9 CHLC0069 (1 male); White Sands National Park, SE edge of dunes, 32.761, -106.189, 1215 m, 11 III 2020, Eric Metzler leg. (1 female); Texas: Culberson County, Sierra Diablo W[ildlife] M[anagement] A[rea], 2 V [19]84, E. Knudson / CNCLEP7661 (1 male). CNC, CSUC, DLWC, JVC, LGC, NMNH.

#### Diagnosis.

*Pooleapsaphidoides* resembles *P.grandimacula* but is usually larger (FW length 14–16 mm compared to 11.5–15 mm) and has a darker charcoal to dark olive gray forewing lacking black streaks; the FW of *P.grandimacula* is medium gray with prominent longitudinal black bars. Pale scales distal to the distal postmedial line in *P.psaphidoides* appear as a distinct white spot in the fold.

**Figure 10.**
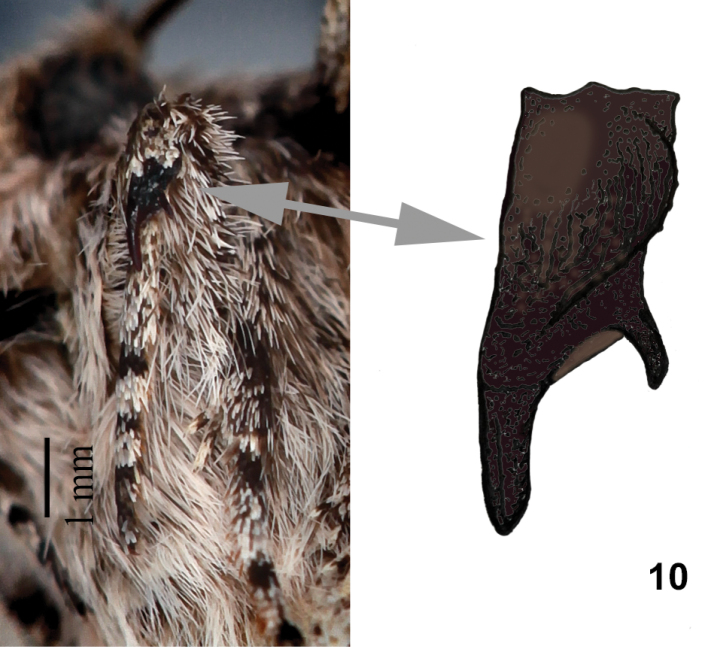
Foretibial claw of *Pooleapsaphidoides* (photograph and drawing).

The male genitalia of *P.psaphidoides* are most easily distinguished from *P.grandimacula* by the position of the clasper ampulla, close to the mid valve in *P.psaphidoides* and near the base of the cucullus in *P.grandimacula*. The cornuti on the vesica of *P.psaphidoides* are longer than those of *P.grandimacula*, forming a group of long stout spines in the proximal band that *P.grandimacula* lacks. Females of *P.psaphidoides* have a large rightward bulge in the anterior ductus bursae, lacking in *P.grandimacula*.

**Figure 11.**
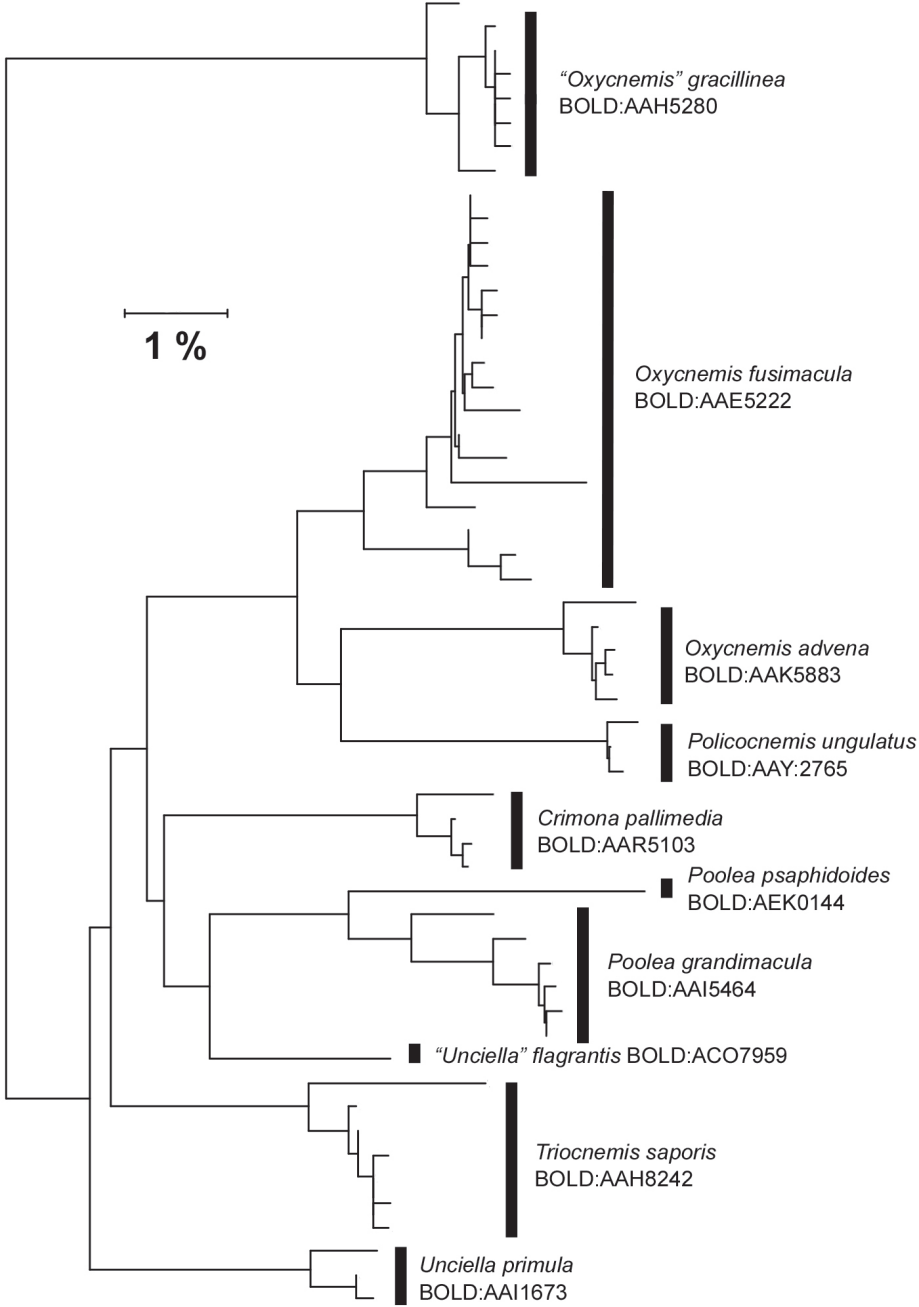
CO1 barcode neighbor-joining tree of North American Triocnemidina sensu Poole (2022): *Crimona*, *Oxycnemis*, *Policocnemis*, *Poolea*, *Triocnemis*, *Unciella*, “*Unciella*.” “*Oxycnemis” gracillinea*, now reassigned to Onconemidinae and placed provisionally in *Sympistis*, is included for comparison.

The DNA barcode BIN of *P.psaphidoides*, BOLD:AEK0144, differs from that of *P.grandimacula*, BOLD:AAI5464, by greater than 4.2% (Fig. 11).

#### Description.

***Adults*.** Dorsal head and thorax charcoal gray. *Head* – As for genus. Antenna scales dark gray. Labial palpus scales mostly dark gray, scattered off-white on segments 2 and 3. Frons scales gray. *Thorax* – Vestiture as for genus; black lines on prothoracic collar and tegula indistinct. *Legs* – As for genus. *Wings*: FW length 14.0–16.0 mm (males); 15.0 mm (female); even charcoal gray, occasionally dark olive, with slightly darker gray medial area; lines and spots as for genus; medial line faint; pale scales abutting postmedial line white in fold; fringe weakly checkered brown gray and charcoal; horizontal black lines absent. HW as for genus, ground pure white. *Abdomen* – As for genus; tufts dark gray. *Male genitalia* – Uncus and juxta as for genus. Juxta height 2× width. Valve as for genus, length 5× width; ampulla arising slightly distal to mid valve. Phallus and vesica as for genus; a cluster of subbasal cornuti thicker and longer than adjacent cornuti; cornuti on mid vesica long and gracile, decreasing gradually in length toward vesica apex. *Female genitalia* – Papilla analis, segment A8, and bursa copulatrix as for genus. Ductus bursae with broad bulge rightward near attachment to corpus bursae.

#### Etymology.

The name refers to the superficial resemblance of this moth to several *Psaphida* species from eastern North America.

#### Distribution and ecology.

**Figure 12.**
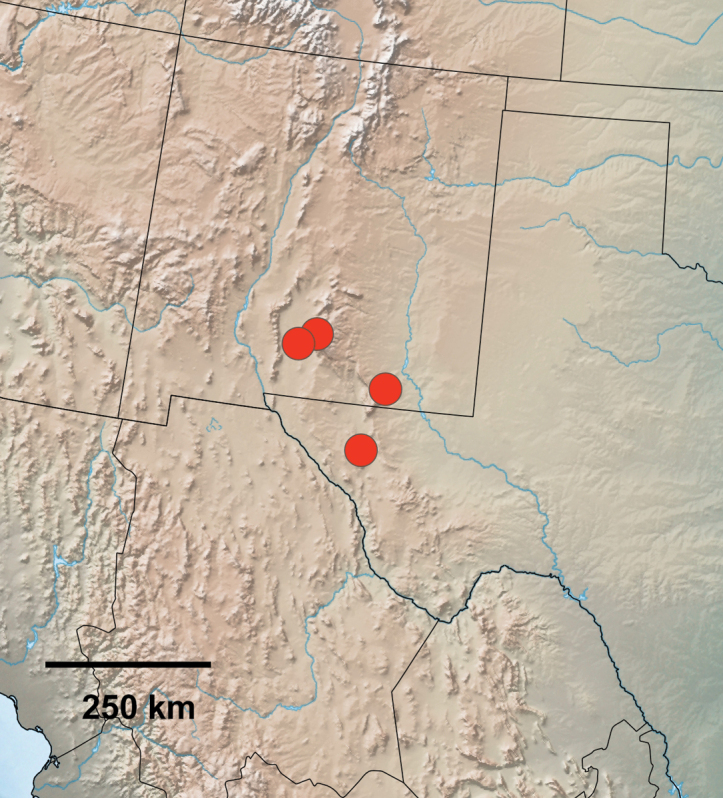
Distribution of examined material of *Pooleapsaphidoides* in New Mexico and Texas, USA.

*Pooleapsaphidoides* has a very limited distribution in the Southwest. It is only known definitively from four localities in southern New Mexico east of the Continental Divide, two each in Otero and Eddy counties, and one location in Culberson County in western Texas (Fig. 12). These localities span approximately 200 kilometers. Two specimens from near the Guadalupe Mountains in Texas called “Triocnemidini New Genus 1 new species 1” by Poole (2022) probably refer to *P.psaphidoides* based on the adult description and illustrated male genitalia but were not examined for this study. The Guadalupe Mountains are located within the known range of the species.

*Pooleapsaphidoides* has been collected in open shrub desert at elevations from 1280 to 1725 meters. The type locality habitat consists of creosote bush [*Larreatridentata* (DC.) Coville, Zygophyllaceae] shrubland with scattered junipers [*Juniperus* sp. (Cupressaceae) and bunchgrasses (Poaceae)]. One female is from gypsum dunes at White Sands National Park, possibly a stray since it is the only specimen of this species found at this locality in over a decade of intensive collecting (E. Metzler pers. comm. 2020). Collection dates are from mid-March to early May suggesting a single brood.

The early stages are unknown.

## ﻿Discussion

*Poolea* and *Oxycnemis* are assigned to Amphipyrinae. No noctuid subfamily has been more difficult to define or seen greater flux—a history reviewed by Keegan et al. (2019). The number of genera in the subfamily has ranged from roughly half of the world’s noctuid fauna when it was first proposed by Hampson in the late nineteenth century (Kitching 1984) to only the type genus a century later (Kitching and Rawlins 1999). In the newly published checklist of North American Lepidoptera north of Mexico (Pohl and Nanz 2023), the subfamily includes 30 genera that are arranged in two tribes.

The last published checklist of Noctuidae for North America north of Mexico prior to the 21^st^ century was that of Franclemont and Todd (1983). Comparing Amphipyrinae in the Franclemont and Todd list to that of Pohl and Nanz (2023) illustrates the massive changes that the subfamily has undergone in North America over four decades. Franclemont and Todd included 106 genera in Amphipyrinae. Fewer than a third of these genera remain in Amphipyrinae currently, and only half (15 of 30) of the amphipyrine genera in Pohl and Nanz were placed in their current tribe or subfamily in 1983.

A satisfactory delimitation of Amphipyrinae has been hampered by a lack of known synapomorphic morphological characters in this and related subfamilies, as well as limited knowledge of the early stages of many species (Keegan et al. 2019). Increased scrutiny of Noctuoidea higher classification around the turn of the last century produced an increasingly precise and cohesive classification of the subfamily, resulting first in its paring down to 73 genera by Lafontaine and Schmidt (2010, 2015). Shortly thereafter Keegan et al. (2019, 2021) probed most of the genera in Amphipyrinae sensu Lafontaine and Schmidt by sequencing multiple genes and corroborating the molecular results with adult morphology and recent discoveries of many larvae and their biology. They showed that the subfamily remained “massively” polyphyletic and reassigned a large number of amphipyrine taxa to other groups, including 10 different subfamilies. A few “amphipyrine” genera were even found to contain species of more than one subfamily, including *Oxycnemis* (Keegan et al. 2019). Despite these works, the subfamily classification remains mostly inferred from nucleotide states and relevant synapomorphies have yet to be defined precisely.

While Keegan et al.’s results provided the much-needed support for a satisfying rational Amphipyrinae classification, Psaphidini subtribes were not specifically addressed in either of their studies as they were poorly supported (Keegan et al. 2021, fig. 4) and beyond the scope of the papers which were focused on proper subfamilial placement of genera. More genetic data will be needed to fully resolve the intratribal relationships in Psaphidini. Nonetheless, the genera placed in Triocnemidina by Poole, along with *Hemigrotellaargenteostriata*, are a sister group to a clade comprised of most of the genera in Psaphidina. Therefore, it is reasonable to follow this subtribe arrangement for the time being.

Although neither of the Keegan et al. studies (2019, 2021) included a *Poolea* species, its assignment to Triocnemidina based on morphological characters is supported strongly by DNA barcodes. *Poolea* consistently groups adjacent to *Crimona*, *Triocnemis*, *Unciella*, and “*Unciella*” on neighbor-joining trees (Fig. 11). *Crimona*, *Triocnemis*, and *Unciella* also form a clade in Keegan et al.’s maximum-likelihood trees. These five genera are also similar structurally, with similar wing shapes, foretibial claws, and male genitalia. The largest structural differences between the adults appear to be in the female genitalia based on a review of the illustrations in Poole (2022).

## Supplementary Material

XML Treatment for Poolea

XML Treatment for Poolea grandimacula

XML Treatment for Poolea psaphidoides

## ﻿Additional information

### Author ORCIDs

Lars G. Crabo https://orcid.org/0009-0004-1089-5511

### Data availability

All of the data that support the findings of this study are available in the main text.
